# Methotrexate Pneumonitis After a Low-Dose Medication Error: A Case Report

**DOI:** 10.7759/cureus.23078

**Published:** 2022-03-11

**Authors:** Maria Manuel Silva, Carla Campinho Ferreira, Maria Afonso Garcia, Edite Pereira

**Affiliations:** 1 Endocrinology, Diabetes and Metabolism, Centro Hospitalar Universitário de São João, Porto, PRT; 2 Serviço de Reumatologia, Hospital de Braga, Braga, PRT; 3 Serviço de Saúde Ocupacional, Hospital Garcia de Orta, Lisbon, PRT; 4 Internal Medicine, Centro Hospitalar Univeristário de São João, Porto, PRT

**Keywords:** methotrexate-induced pneumonitis, methotrexate-induced pancytopenia, rheumatoid arthritis, methotrexate toxicity, oral methotrexate

## Abstract

Methotrexate is recommended as the first choice of standard drug therapy following the diagnosis of rheumatoid arthritis. Pneumonitis related to methotrexate is a serious, unpredictable adverse event that may become life-threatening. We reported a case of a 68-year-old woman with rheumatoid arthritis that misunderstood the directions for use and took methotrexate daily, instead of weekly, leading to hepatic, hematological, and pulmonary toxicity.Although the histological evaluation was not performed, patient’s clinical presentation, in addition to subsequent investigational findings, supported a diagnosis of pneumonitis resulting from MTX exposure. Toxic dosing over a long period of time along with the concomitant taking of pantoprazole and hypoalbuminemia could have increased the incidence of some adverse events. Concerning pneumonitis related to methotrexate, the toxic dose may have accelerated the pulmonary manifestations, but we do not know if correct dose had been taken, this adverse event would occur. This case enlightened two important issues in rheumatoid arthritis treatment: the possibility of medication errors and the rare, but potentially life-threatening, methotrexate-induced pneumonitis. Improving education and warnings when prescribing and dispensing low-dose methotrexate is essential.

## Introduction

Methotrexate (MTX) is recommended as the first choice of standard drug therapy following the diagnosis of rheumatoid arthritis (RA) [1,2]. The most common adverse events associated with MTX are gastrointestinal, hematologic, and hepatic [1,2]. Pneumonitis related to MTX (MTX-pneumonitis) is a serious, unpredictable adverse effect that occurs in 3-5% of RA patients and may become life-threatening [3,4].

In the past years, frequent medication errors related to low-dose MTX regimens have been reported. These could lead to MTX toxic doses, severe adverse events, and death [5-9]. This article was previously presented as a poster at the VII Congresso Nacional de Autoimunidade - XXVI Reunião Anual do Núcleo de Estudos de Doenças Auto-imunes on June 15, 2021.

## Case presentation

A 68-year-old woman presented to the hospital with a three-week history of bullous and ulcerative lesions in oral mucosa, odynophagia, and progressive asthenia. Her medical history included RA (diagnosed two years ago), fibromyalgia, dyslipidemia, overweight, and depressive disorder. The patient denied a past history of pulmonary disease. Her drug therapy included tramadol 100 mg daily, gabapentin 400 mg daily, cyclobenzaprine hydrochloride 10 mg daily, pantoprazole 40 mg daily, sertraline 50 mg daily, clonazepam 2 mg daily, and fenofibrate 267 mg daily. Oral MTX was started 35 days before at a 7.5 mg daily dose, approximately 0.15 mg/kg per day (prescription was 7.5 mg per week, but patient misunderstood the dosing scheme and took it every day), as well as deflazacort at a 30 mg daily dose. Folic acid supplementation and *Pneumocystis jirovecii* prophylaxis was not initiated.

**Figure 1:**
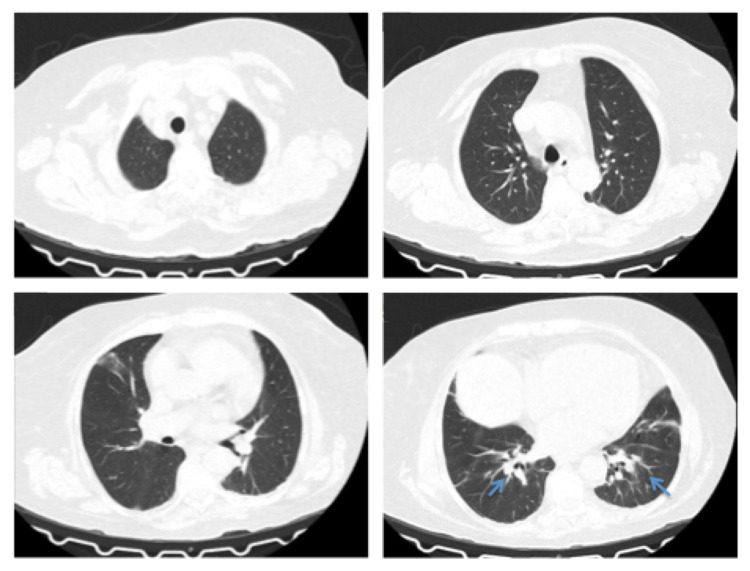
Chest CT at admission. Chest CT scan at admission showed bilateral infiltrates in lower lobes and subpleural in the lingula.

On examination, she was prostrated, icteric, and afebrile, her blood pressure was 138/66 mmHg, with a heart rate of 82 beats/min. Blood gas analysis (FiO_2_ of 21%) showed mixed respiratory failure with respiratory academia-pH of 7.318, PCO_2_ of 47.5 mmHg, PO_2_ of 56.5 mmHg, HCO_3_ 23.8 mEq/L, and SaO_2_ at 86.6%. A respiratory rate was not available. Blood tests showed leukopenia (0.49×10^9^/L), anemia (hemoglobin 9.8 g/dL), hypoalbuminemia (30.6 g/L, normal range: 38-51 g/L), increased C-reactive protein (464.8 g/L, normal range: <3.0 g/L), aspartate aminotransferase (67 U/L, normal range: 10-31 U/L), total bilirubin (1.79 mg/dL), and conjugated (1.20 mg/dL). Creatinine was 0.88 mg/dL (Chronic Kidney Disease Epidemiology Collaboration estimate glomerular filtrate rate (CKD-EPI eGFR) = 72 mL/min/1.73 m²) and urea was 41 mg/dL (normal range: 10-50 mg/dL). MTX serum level was 0.02 μmol/L, but we didn’t know the time of the last MTX intake. A chest X-ray revealed a diffuse interstitial pattern. Computed tomography (CT) of the chest demonstrated discrete infiltrates predominantly peribronchovascular in both the lower lobes and subpleural in the lingula. No lymphadenopathy was observed (Figure 1). She did not have a previous chest X-ray/CT to compare. Blood and urine cultures were collected. Bronchoalveolar lavage (BAL) samples were collected, demonstrating low cellularity and neutrophils. Samples were sent to microbiological analysis. *Streptococcus pneumoniae* urine antigen test was positive. The treatment regimen was piperacillin-tazobactam, vancomycin, ceftazidime, fluconazole therapy, and methylprednisolone at 2 mg/kg/day dose. Coverage for *Pneumocystis jirovecii* was not done because chest CT images were not the most typical (did not demonstrate extensive ground-glass opacities or cystic lesions), although this agent needs to be considered in immunocompromised individuals.

**Figure 2:**
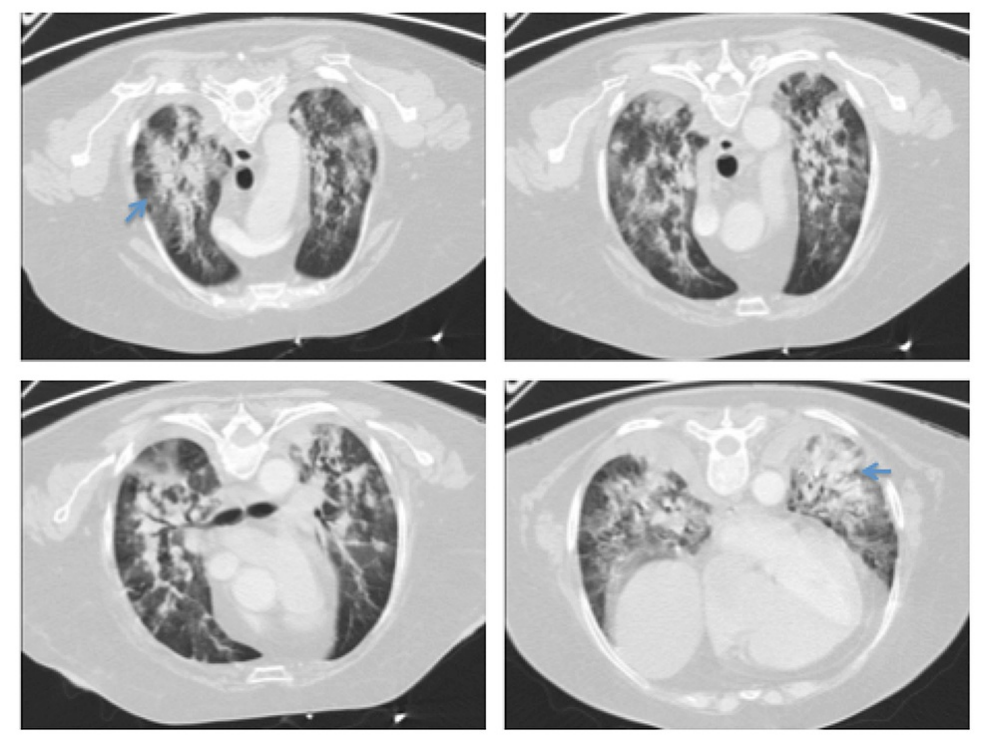
Chest CT 24 hours after admission. Chest CT scan after 24 hours of admission showed bilateral pleural effusion, with signs of alveolar consolidation centered on perihilar topography with symmetrical distribution.

After 24 hours, the hypoxic respiratory insufficiency and hematological parameters aggravated and the patient needed ventilatory support with invasive mechanical ventilation, red cell concentrates, and platelets transfusion being transferred to an intensive care unit. Chest CT was repeated and revealed bilateral pleural effusion, with signs of alveolar consolidation centered on perihilar topography with symmetrical distribution (Figure 2). The treatment regimen with piperacillin-tazobactam, vancomycin, ceftazidime, fluconazole therapy, and methylprednisolone were maintained. Ten days after initiating invasive ventilation, the patient’s condition improved allowing the switch to non-invasive ventilation. The blood and urine cultures were negative. BAL cultures were negative. Also, the *Pneumocystis jirovecii* search in BAL was negative. Gradually, her pulmonary dynamic was improved as well as the mucositis and pancytopenia.

The patient’s clinical presentation, in addition to subsequent investigational findings, supported a diagnosis of pneumonitis resulting from MTX exposure. She initiated physical and respiratory rehabilitation and 17 days after admission she was discharged to an internal medicine nursery to continue rehabilitation. The patient remained in the internal medicine nursery for 21 days, having undergone motor rehabilitation and respiratory kinesitherapy. Due to the general improvement, she was transferred to a rehabilitation unit. Currently, the patient is only followed up in rheumatology appointments. MTX therapy was never restarted. The patient was not treated for RA for up to two years after this admission. Because of arthralgias in small joints of both hands, she started therapy with sulfasalazine and prednisolone. Presently, she is under sulfasalazine at 1500 mg daily dose and prednisolone 5 mg daily without any complaint or mobility restriction. Concerning respiratory complaints, apparently she did not have any respiratory sequelae or complaints. Chest CT was not repeated.

## Discussion

MTX is a folic acid analog that competitively inhibits folic acid reductase, exerting antiproliferative and immunomodulating effects [1,2]. In the standard initial treatment after the diagnosis of RA, MTX should be used if it is not contraindicated [2,10]. For rheumatic diseases’ treatment, MTX can be administered weekly by different routes: oral, subcutaneous, or intramuscular [11]. Oral MTX is widely preferred because of its low costs and patient preferences [11]. To minimize acute and chronic toxicity, MTX should be escalated to a weekly dose of about 0.3 mg/kg, and this escalation should be done within four-six weeks [2]. A dose of 30 mg per week is, commonly, considered the highest tolerable dose of prolonged oral MTX treatment [11,12]. The most common adverse events in this low-dose regimen are gastrointestinal (nausea, stomach upset, and loose stools), hematologic (particularly macrocytosis), and hepatic (mild elevations in hepatic transaminases). Severe adverse events (myelosuppression, pneumonitis, opportunistic infection, and lymphoproliferative disease) are more common with higher doses of MTX and elderly patients [1,13]. Moreover, some conditions such as hypoalbuminemia, renal dysfunction, and certain concomitant medications, including nonsteroidal anti-­inflammatory drugs and proton pump inhibitors, increase a patient’s risk of developing toxic effects from MTX [14].

MTX-pneumonitis is a serious and unpredictable adverse event with an incidence that ranges between 0.3 and 11.6%, depending on the methodology and criteria applied. It has an acute, subacute, or even chronic type of presentation but may become life-threatening [3]. It is usually observed within the first year of treatment [3,4]. Its histology demonstrates an interstitial inflammation and fibrosis [3]. A different entity is interstitial lung disease associated with RA (ILD-RA). The differential diagnosis between these two could be difficult because they share the same clinical manifestations. In a 2019 review, Fragoulis et al. proposed an algorithm to help clinicians in these diagnoses [4]. According to this, in the setting of recent MTX initiation, MTX-pneumonitis is always a concern, especially if the onset of symptoms is acute or subacute. If the onset is more insidious, ILD-RA is a possibility [4]. Also, it is important to rule out other possible diagnoses, like pulmonary infection [3]. The histological and imaging findings are another criteria that could help the diagnosis [3,4]. In our patient, a lung biopsy was not performed. Although histological evaluation was not performed, clinical, microbiological, and imaging features could exclude infection and other pulmonary diseases, being achievable to determine a possible MTX-pneumonitis etiology. In MTX-pneumonitis, radiological findings reflect the underlying histopathologic process and include mostly non-specific interstitial pneumonia, more so than bronchiolitis obliterans organizing pneumonia. Therefore, on CT scanning, scattered or diffuse ground-glass opacities are seen in the early stages and basal fibrosis in the later stages of the disease [4].

Another important issue addressed in this case is the possibility of medication errors with MTX therapy. In the past years, frequent medication errors have been reported, leading to toxic doses, serious adverse events, and death [5-9]. Based on large series, many countries such as France [6], Australia [9], Denmark [15], Spain [7], and the USA [5] mentioned a dramatic death rate after MTX dosing errors that could range from 5.2% to 24%. The causes and locations of the errors depend on the countries [6]. Overall, patient and erroneous prescription renewal by a non-specialist physician are the most common causes for medication errors [5-7,9,15]. In this case, the error was made by the patient who did not understand the dosing scheme. Some aspects augment the probability of toxic events, such as patient’s renal function impairment, advanced age, and concomitant medication [5,6,13,15]. In this case, our patient was taking pantoprazole, a proton pump inhibitor, which is related to a higher incidence of toxic events [13]. Additionally, on admission, the patient had hypoalbuminemia, which can also increase the incidence of toxic events [13]. Following an accumulating number of MTX medication errors, in 2019, the European Medicines Agency recommended new measures to prevent serious and potentially fatal errors with the dosing of MTX in inflammatory diseases like RA [16]. The new measures include restricting who can prescribe this medicine, making warnings on the packaging more prominent, and providing educational materials for patients and healthcare professionals [16].

Probably, if the correct dosage was taken the hematological, hepatic, and mucositis adverse events would not have occurred. Also, our patient took an erroneous dose for 35 days. In a recent study based on French poison control and pharmacovigilance centers, the mean duration of the error was 11.7 ± 12.2 days (range: 2 to 90). However, in this study, severe outcomes or death were associated with a short-term error. [6]. Regarding MTX-pneumonitis, the toxic dose may have accelerated the pulmonary manifestations, but we do not know if the correct dose had been taken, this adverse event would occur.

## Conclusions

This case enlightened two important issues in RA treatment: the possibility of medication errors and the rare, but serious, pulmonary side effects associated with MTX. It is important to improve education and warnings when prescribing and dispensing low-dose MTX. Therefore, prescribers should ensure that the patient and/or their caregiver clearly understand the dosing scheme.
